# Apocynin: Chemical and Biophysical Properties of a NADPH Oxidase Inhibitor

**DOI:** 10.3390/molecules18032821

**Published:** 2013-03-01

**Authors:** Maicon S. Petrônio, Maria Luiza Zeraik, Luiz Marcos da Fonseca, Valdecir F. Ximenes

**Affiliations:** 1 Departamento de Análises Clínicas, Faculdade de Ciências Farmacêuticas, Unesp-Univ Estadual Paulista, Araraquara, SP 14801-902, Brazil; E-Mails: petronioms@fcfar.unesp.br; fonseclm@fcfar.unesp.br; 2 Departamento de Química Orgânica, Instituto de Química, Unesp-Univ Estadual Paulista, Araraquara, SP, 14800-900, Brazil; E-Mail: zeraik.marialuizaze@gmail.com; 3 Departamento de Química, Faculdade de Ciências, Unesp-Univ Estadual Paulista, Bauru, SP 17033-360, Brazil

**Keywords:** NADPH oxidase, apocynin, hydrogen peroxide, albumin, binding constant

## Abstract

Apocynin is the most employed inhibitor of NADPH oxidase (NOX), a multienzymatic complex capable of catalyzing the one-electron reduction of molecular oxygen to the superoxide anion. Despite controversies about its selectivity, apocynin has been used as one of the most promising drugs in experimental models of inflammatory and neurodegenerative diseases. Here, we aimed to study the chemical and biophysical properties of apocynin. The oxidation potential was determined by cyclic voltammetry (Epa = 0.76V), the hydrophobicity index was calculated (logP = 0.83) and the molar absorption coefficient was determined (ε_275nm_ = 1.1 × 10^4^ M^−1^ cm^−1^). Apocynin was a weak free radical scavenger (as measured using the DPPH, peroxyl radical and nitric oxide assays) when compared to protocatechuic acid, used here as a reference antioxidant. On the other hand, apocynin was more effective than protocatechuic acid as scavenger of the non-radical species hypochlorous acid. Apocynin reacted promptly with the non-radical reactive species H_2_O_2_ only in the presence of peroxidase. This finding is relevant, since it represents a new pathway for depleting H_2_O_2_ in cellular experimental models, besides the direct inhibition of NADPH oxidase. This could be relevant for its application as an inhibitor of NOX4, since this isoform produces H_2_O_2_ and not superoxide anion. The binding parameters calculated by fluorescence quenching showed that apocynin binds to human serum albumin (HSA) with a binding affinity of 2.19 × 10^4^ M^−1^. The association did not alter the secondary and tertiary structure of HSA, as verified by synchronous fluorescence and circular dichroism. The displacement of fluorescent probes suggested that apocynin binds to site I and site II of HSA. Considering the current biomedical applications of this phytochemical, the dissemination of these chemical and biophysical properties can be very helpful for scientists and physicians interested in the use of apocynin.

## 1. Introduction

Nicotinamide adenine dinucleotide phosphate oxidase (NADPH oxidase, NOX) is a multicomponent enzyme system expressed in many cell types that catalyzes the one-electron reduction of molecular oxygen to the free radical species superoxide anion [1,2,3]. Since its characterization as a NOX assembly inhibitor in 1994 [4], apocynin (4-hydroxy-3-methoxyacetophenone) has been used as one of the most promising drugs in experimental models of disease in which the involvement of reactive oxygen species (ROS) is well-established; for instance, in vascular, inflammatory and neurodegenerative pathologies. Hence, despite its structural simplicity as a derivative of acetophenone and controversies regarding its potency and selectivity as a NOX inhibitor [5,6,7,8,9], the pharmacological effects that have been obtained using this phytochemical are quite striking. For example, the administration of apocynin in mice deficient in the LDL receptor showed decreased P-selectin expression, VCAM-1 expression, platelet adhesion, aortic elastic modulus and reduced total monocyte accumulation in a dose-dependent manner [10]. Apocynin prevented microglial activation induced by oligomeric amyloid-β, suggesting that NADPH oxidase activation is involved in the effects of this neurotoxin [11]. Apocynin prevented increased ROS generation and arterial stiffness in deoxycorticosterone acetate-salt-induced hypertension in rats [12]. Diapocynin, a dimeric oxidized form of apocynin, had profound neuroprotective effects in a pre-clinical animal model of Parkinson’s disease by attenuating oxidative damage and neuroinflammatory responses [13]. Apocynin ameliorated diabetes-related erectile dysfunction by reducing ROS production in experimental models of diabetic rats [14]. Depression in a stress-induced animal model provoked an increase in the expression of the cytosolic components of NADPH oxidase, p47phox and p67phox, in the brains of rats; however, the pharmacological inhibition of NADPH oxidase by apocynin during the stress or post-stress period completely blocked depressive behavior [15]. The inhalation of apocynin reduced the concentration of hydrogen peroxide in the exhaled breath condensates of chronic obstructive pulmonary disease patients [16].

Apocynin was originally extracted from the root extracts of the medicinal herb *Picrorhiza kurroa*, from the Himalayan Mountains; however, nowadays apocynin is an inexpensive substance and can be obtained commercially from several companies, which facilitates the broad application of this relatively non-toxic phytochemical. It is accepted that the intracellular mechanism of NADPH oxidase inhibition by apocynin is related to the assembly of this multicomponent enzyme complex by blocking the translocation of the cytosolic fractions p47-phox and p67-phox to the membrane fraction [4,17]. Moreover, increasing evidence has demonstrated that apocynin also inhibits the expression of NOX components such as p47-phox, p67-phox and gp91-phox [18,19]. Apocynin seems to act as a prodrug, since it must be initially oxidized into its dimeric form, diapocynin, the supposedly active form of apocynin [4,17]. Corroborating this, diapocynin was isolated when apocynin was incubated with activated neutrophils [20], and purified diapocynin and its oligomers have been demonstrated as bring more effective than apocynin itself in several experimental studies [13,21,22]. For these reasons and considering the relevance and high applicability of apocynin, here we aimed to study the chemical and biophysical characteristics of apocynin. We believe that this information will be very helpful for scientists interested in the use of apocynin.

## 2. Results and Discussion

**Figure 1:**
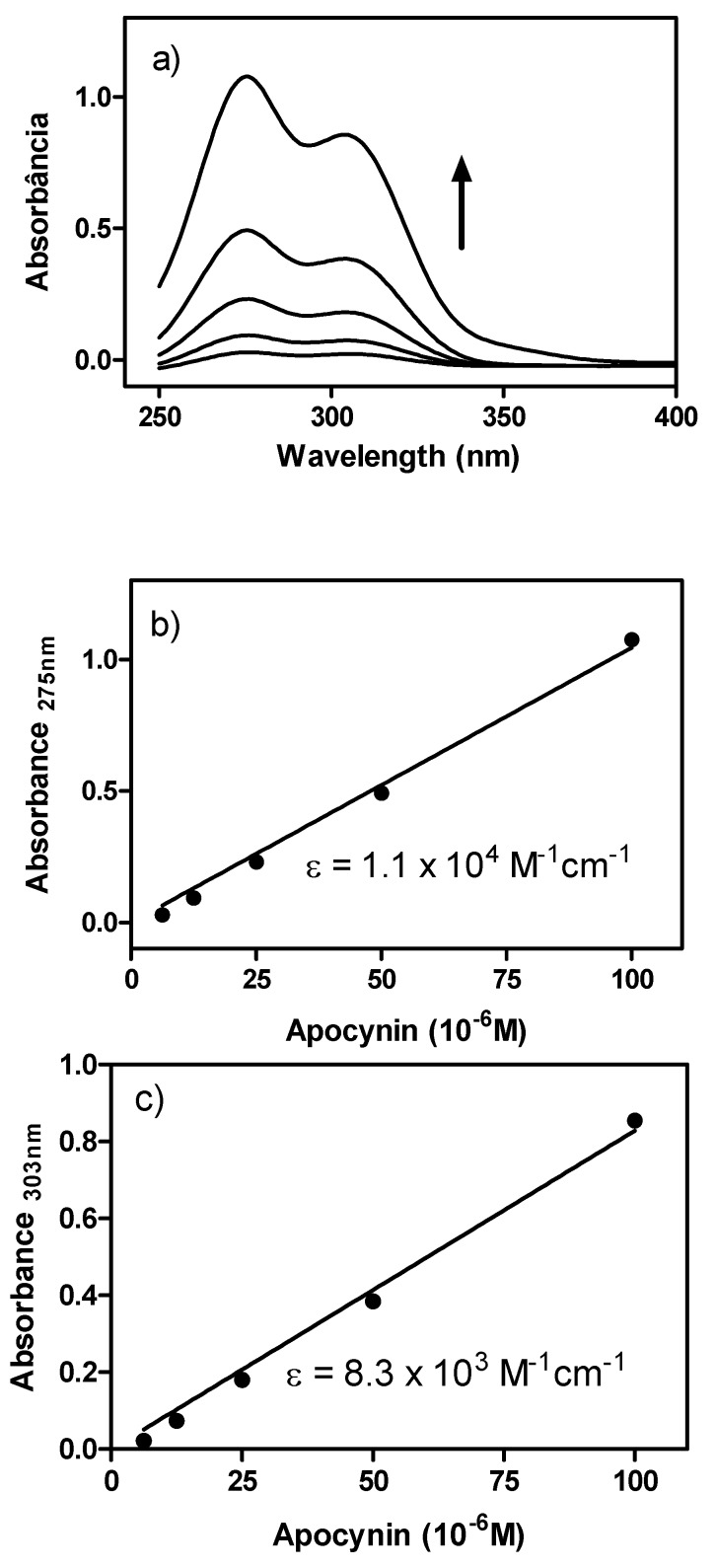
UV-Vis spectrum of apocynin and molar absorption coefficients. (**a**) UV-Vis spectra of apocynin dissolved in water (6.25–100 μM); (**b**) Linear regression at 275 nm, r^2^ = 0.9994; (**c**) Linear regression at 303 nm, r^2^ = 0.9989.

The increasing use of apocynin as an NADPH oxidase inhibitor in numerous *in vitro* and *in vivo* studies [10,11,12,13,14,15,16] was our motivation for this work, where the physical, chemical, spectroscopic and biophysical properties of this compound were investigated. Apocynin has a strong UV-Vis absorption (Figure 1), with two peaks at 275 and 303 nm. The molar absorptivity coefficients were high enough to be used for the quantitative analysis of aqueous apocynin solutions, with detection limits in the micromolar range (Figure 1).

**Figure 2:**
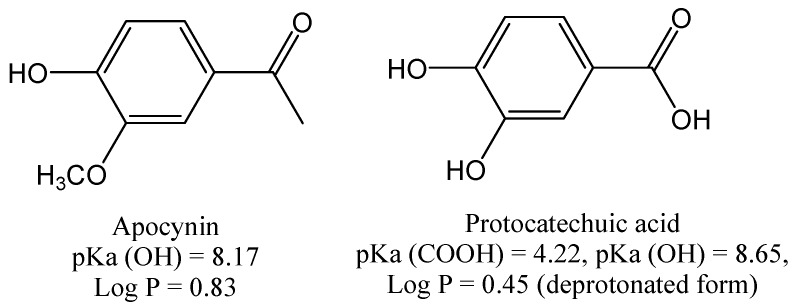
Molecular structures of apocynin and protocatechuic acid and their hydrophobicity indexes (log P) and acidity constants (pKa).

Besides acting as an inhibitor of NADPH oxidase, apocynin is also considered a ROS scavenger [8] and, conversely, a pro-oxidant substance [5,6]. Hence, we studied and compared its redox properties with a structurally similar phenolic compound, protocatechuic acid, which is found in green tea and is recognized for its excellent antioxidant properties [23], but is not used as an NADPH oxidase inhibitor. The structures of these molecules are presented in Figure 2. As can be seen, both compounds have a catechol moiety, the oxidizable part of these molecules; however, in apocynin, one hydroxyl group is alkylated. As a benzoic acid derivative, protocatechuic acid (pKa = 4.22) [24] is ionized at physiological pH. On the other hand, as a phenol derivative, apocynin is relatively less acid (pKa = 8.17) and it will be only slightly ionized at physiological pH (about 10%, calculated using the Henderson-Hasselbalch equation). Another important difference between these molecules is their hydrophobicities, measured as log P values (partitioning coefficient in *n*-octanol/water) [25]. Hence, taking into account their values, it can be assumed that apocynin (log P = 0.83) will have greater access to the cell membrane and intracellular lipophilic sites than protocatechuic acid (log P = 0.45, calculated for its ionized form).

**Figure 3:**
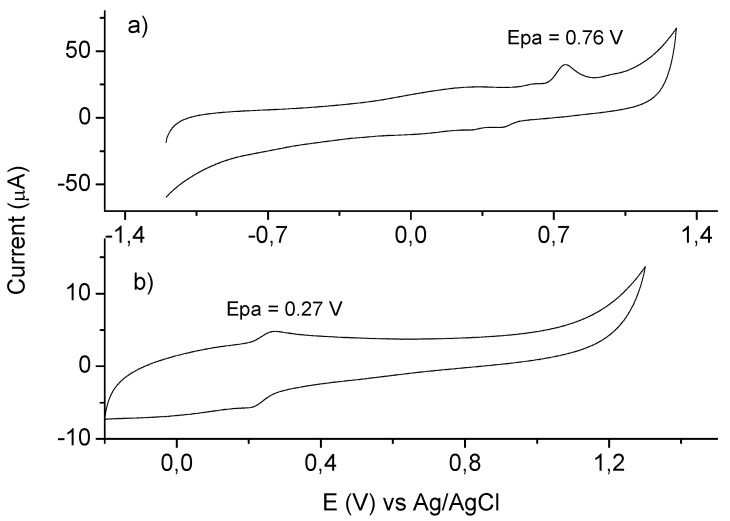
Cyclic voltammograms for apocynin (**a**) and protocatechuic acid (**b**) (0.1 mM) obtained in 0.2 M sodium phosphate buffer at pH 7.0. The scan rate was 5 mV s.

Apocynin was investigated and compared with protocatechuic acid by cyclic voltammetry using a glassy carbon working electrode. The overall cyclic voltammograms of these compounds, obtained at pH 7.0, showed a well-defined anodic wave peaking at 0.76 V and 0.27 V for apocynin and protocatechuic acid, respectively (Figure 3). The higher oxidation potential for apocynin compared to protocatechuic acid is an indication of its lower capacity as a free radical scavenger, which was confirmed in the next experiments.

**Figure 4:**
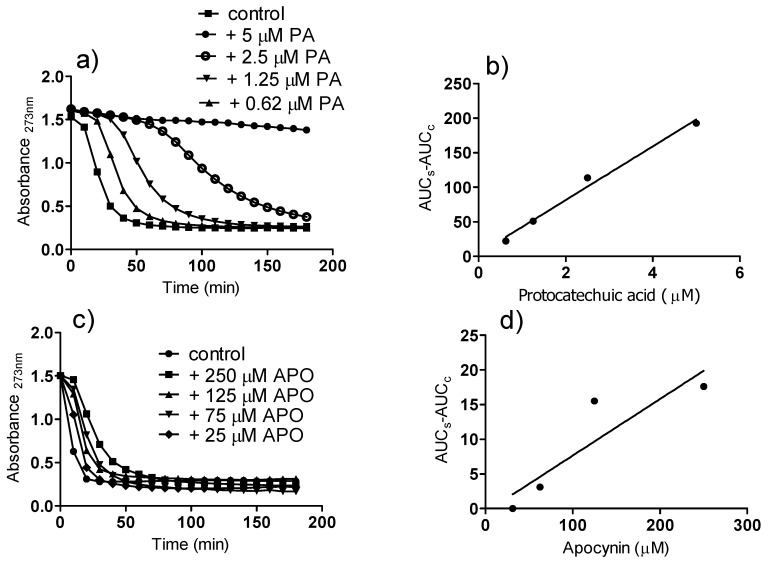
Reactivity of apocynin (APO) and protocatechuic acid (PA) with peroxyl radical (ROO•). (**a**,**c**) Bleaching of the triene (eleostearic acid) by ROO• and the lag phase provoked by the addition of the tested substances. (**b**,**d**) AUC versus concentration.

The results depicted in Figure 4 show the peroxyl radical (ROO•) scavenging capacity of apocynin and protocatechuic acid. In this experimental model, ROO• generated by the thermolysis of AAPH is able to degrade the conjugated triene in eleostearic acid, the major component of tung oil [26]. The oxidation of the triene was monitored by the bleaching of absorption at 273 nm. The addition of the test compounds, by scavenging ROO•, delayed bleaching and produced a concentration-dependent lag phase. As can be seen, protocatechuic acid was significantly more efficient than apocynin as a scavenger of ROO•. The slopes (AUC/concentration) were 38.85 (r^2^ = 0.9935) and 0.081 (r^2^ = 0.8969) for protocatechuic acid and apocynin, respectively.

**Figure 5:**
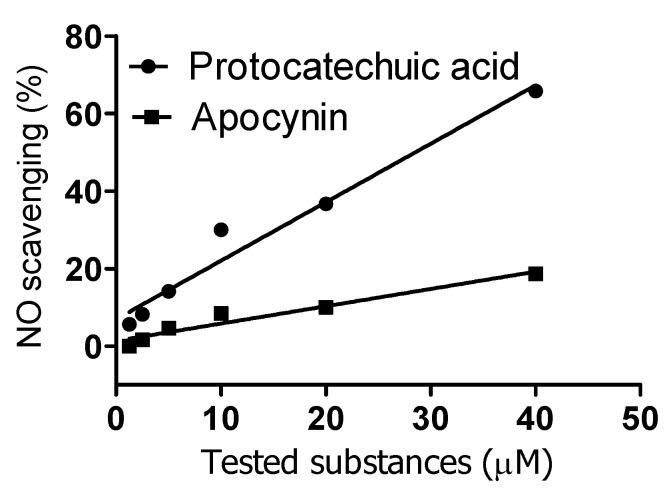
Reactivity of apocynin (APO) and protocatechuic acid (PA) with Nitric oxide. The reaction mixtures were constituted of 10 mM sodium nitroprusside and various concentration of the tested substances in 10 mM PBS, pH 7.4. The production of NO was measured by the Griess method.

The antiradical capacity of apocynin and protocatechuic acid was also compared regarding their efficiency as scavengers of the stable free radical DPPH. Corroborating the previous experiment, apocynin (IC_50_ = 9.7 mM) was a significantly weaker scavenger than protocatechuic acid (IC_50_ = 0.022 mM). Finally, they were compared as scavengers of nitric oxide (NO•), which was generated by decomposition of sodium nitroprusside and measured as nitrite using the Griess reagent. From the results in Figure 5, it can be concluded that apocynin was also not effective as a scavenger of NO• when compared with protocatechuic acid. Altogether, these findings highlight the poor capacity of apocynin as a free radical scavenger and are in agreement with its absence of reactivity with superoxide radical anion [27].

**Figure 6:**
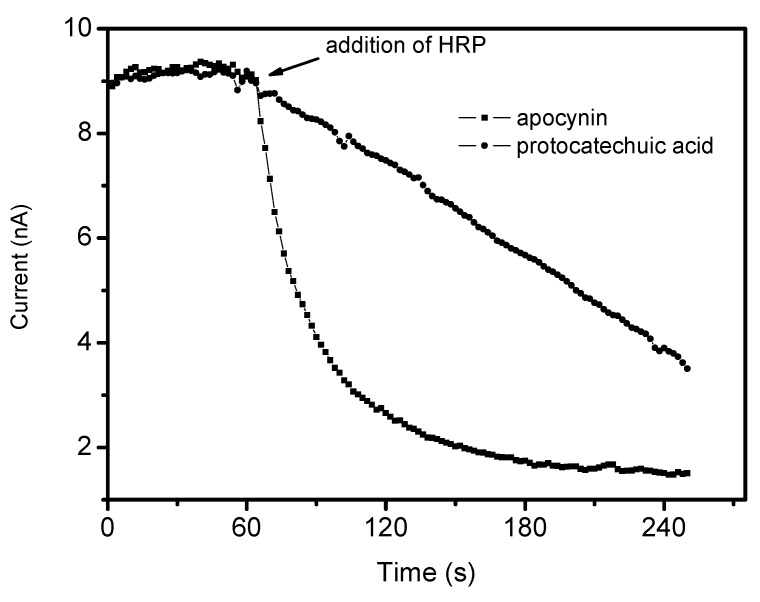
Reactivity of apocynin and protocatechuic acid with hydrogen peroxide. The reaction mixtures were constituted of 1 mM tested substances, 1 mM H_2_O_2_ and 100 nM HRP in 0.1 M PBS, pH 7.4, at 25 °C.

Although a weak free radical scavenger, apocynin reacted promptly with the non-radical reactive species H_2_O_2_ in a reaction catalyzed by peroxidase (Figure 6). In these experiments, the reactions were monitored by amperometry using an H_2_O_2_ selective-electrode. As can be observed, apocynin was completely unreactive with H_2_O_2_; however, the addition of a catalytic amount of peroxidase (HRP) caused an instantaneous consumption of H_2_O_2_ (1 mM in less than 2 min). These results might have important biological implications, since the pharmacological effects of apocynin could be also related to its capacity as a scavenger of H_2_O_2_ and not only the inhibition of its production through the inhibition of NADPH oxidase. However, the necessity of peroxidase as a catalyst must be emphasized; hence, apocynin can act as direct scavenger of this endogenous oxidant only in cells endowed with peroxidases. It is also worth noting that, in contrast to the reactions with radical species, the consumption of H_2_O_2_ catalyzed by peroxidase was more efficient using apocynin than protocatechuic acid.

**Figure 7:**
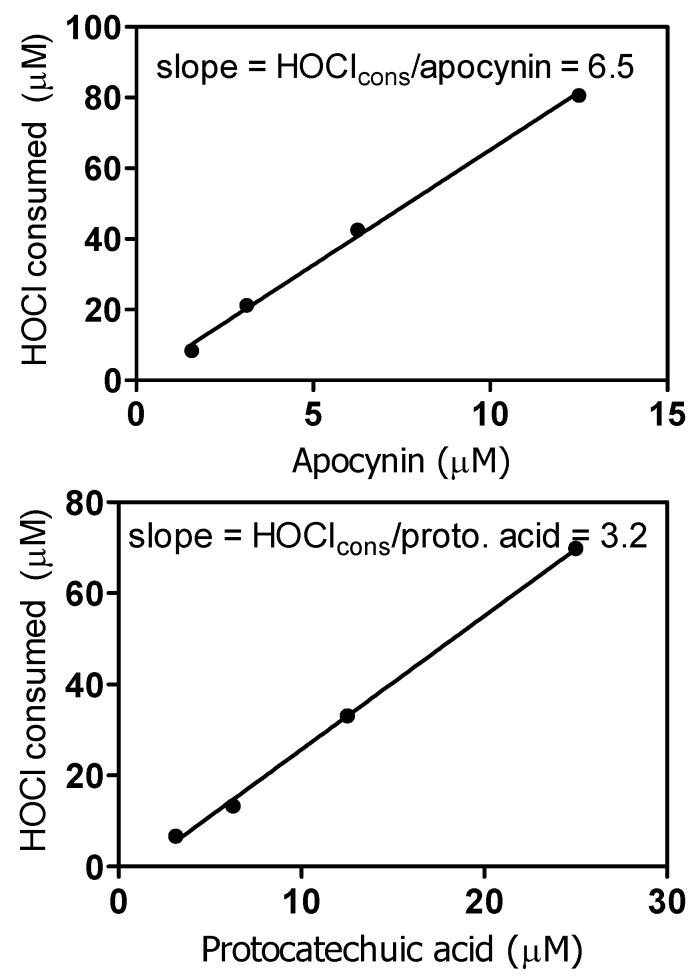
Reactivity of apocynin and protocatechuic acid with hypochlorous acid. The reaction mixtures were constituted of 100 μM HOCl and various concentrations of the tested substances in 0.01 M PBS, pH 7.4, at 25 °C. The consumed HOCl was measured using the taurine-chloramine/TMB assay.

As previously demonstrated, apocynin and protocatechuic acid are not effective inhibitors of the neutrophil peroxidase myeloperoxidase (MPO), the enzyme responsible for the production of HOCl [28,20]. However, here we found that apocynin was an excellent scavenger of HOCl. From the results depicted in Figure 7, a stoichiometric relationship of one molecule of apocynin to approximately 6.5 molecules of HOCl was obtained. In agreement with H_2_O_2_ scavenging capacity, apocynin was also more effective than protocatechuic acid.

**Figure 8:**
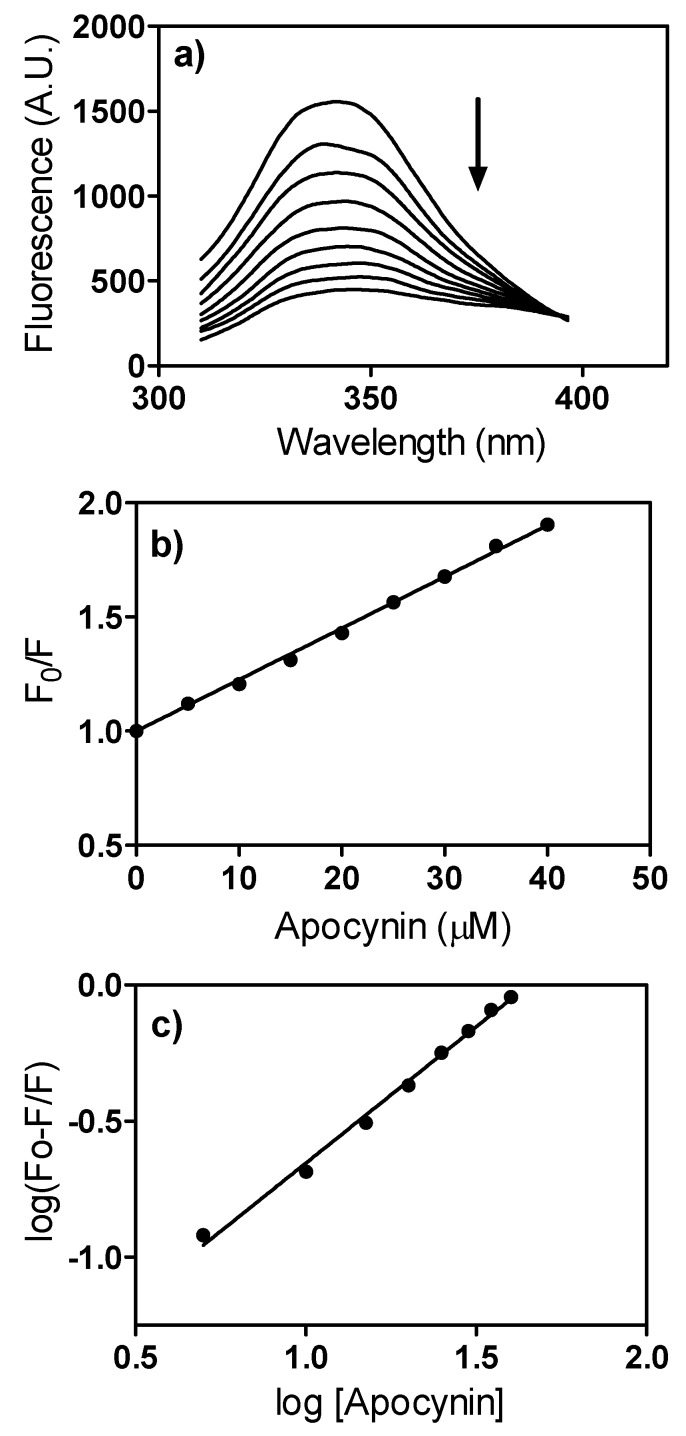
Fluorescence quenching of HSA by apocynin. (**a**) Emission spectra of HSA (1 μM) (λ_ex_ = 295 nm) in the presence of apocynin (0–40 μM). The arrow indicates the addition of apocynin; (**b**) Stern-Volmer plot; (**c**) Plot for apparent binding constant determination.

In many *in vivo* studies, apocynin has been administered orally [13,14,15,29]. Hence, it is important to know how this molecule is transported in the body. For this reason, we studied the interaction of apocynin with human serum albumin, the most abundant protein in the circulatory system and the main protein responsible for the transport of endogenous and exogenous compounds by reversible binding [30]. As an analytical parameter for assessing binding capacity, we used fluorescence quenching, a phenomenon associated with a decrease in the quantum yield of a fluorophore (albumin in this study) caused by collisional deactivation between the fluorophore and the quencher (apocynin in this study) via formation of a ground state complex, among other mechanisms [31]. As can be seen from Figure 8a, the fluorescence intensity of HSA decreased with the addition of apocynin. From these experiments, the quenching constant was calculated by fitting the experimental values to the Stern-Volmer Equation (1):

F_0_/F = 1 + K_sv_*[Q] = 1+ k_q_*τ_0_*[Q] (1)

where F_0_ and F are the steady-state fluorescence in the absence and presence of the quencher, respectively; K_sv_ is the Stern-Volmer constant; k_q_ is the bimolecular quenching constant; τ_0_ is the average lifetime of the fluorophore in the absence of the quencher and [Q] is the concentration of the quencher [31]. As can be seen from Figure 8b, excellent linearity (r^2^ = 0.9979) was obtained and a K_sv_ of 2.25 × 10^4^ M^−1^ was calculated. From this value and assuming τ_0_ for albumin as 10^−8^ s [32], the k_q_ resulted in 2.25 × 10^12^ M^−1^ s^−1^. As this value is higher than the maximum scatter collision quenching constant (2 × 10^10^ M^−1^ s^−1^) [33], the quenching mechanism can be considered a static process; in other words, quenching is caused by the formation of a ground state complex between apocynin and HSA. Considering this, the apparent binding constant (K_a_) between apocynin and HSA could be determined using Equation (2):

log [(F_0_ − F)/F] = log K_a_ + n log [Q] (2)

From the linear fit of log [(F_0_−F)/F] versus the concentration of apocynin to the equation, a K_a_ value of 2.19 × 10^4^ M^−1^ was obtained and “n”, the number of binding sites, was calculated as 1.002; an indication that there is only one association between apocynin and HSA (Figure 8c). It is important to note that the obtained value of the apparent binding constant has the same magnitude compared to several molecules which are able to bind to has; for instance, ascorbic acid K_a_ = 2.24 × 10^4^ M^−1^ [34], salvinolic acid K_a_ = 7.36 × 10^4^ M^−1^ [35] and naphazoline K_a_ = 2.60 ×10^3^ M^−1^ [36]. Hence, we can assume that apocynin can be transported by albumin.

**Figure 9:**
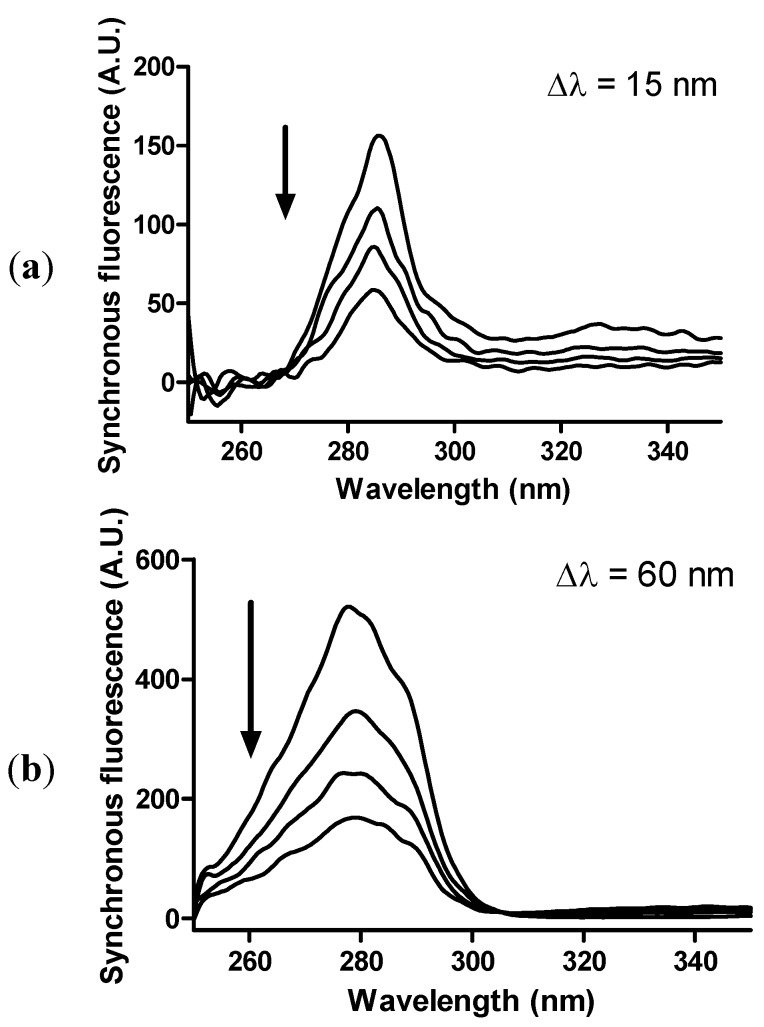
Effect of apocynin on HSA synchronous fluorescence. (**a**) Quencher of synchronous fluorescence (scanning at fixed Δλ = 15 nm). (**b**) Quencher of synchronous fluorescence (scanning at fixed Δλ = 60 nm). The mixtures consisted of 1.0 μM HSA and apocynin (0–30 μM) in 10 mM PBS, pH 7.2 at 25 °C. The arrow indicates the addition of apocynin.

The binding of apocynin to albumin could provoke alterations in the tridimensional structure of the protein. To evaluate this possibility, we used synchronous fluorescence, a technique in which the excitation and emission wavelengths are scanned simultaneously at a fixed wavelength increment (Δλ). It is well-established that synchronous fluorescence spectra provide information on the microenvironment in the vicinity of the fluorophore functional group. For proteins, Δλ can be fixed at 15 or 60 nm and the results will reflect the alteration in the microenvironment next to tyrosine or tryptophan residues, respectively [37]. As depicted in Figure 9a,b, despite the quenching effect, binding to apocynin did not provoke significant alterations in the profile of synchronous fluorescence; hence, we can conclude that apocynin did not alter the structure of albumin.

**Figure 10:**
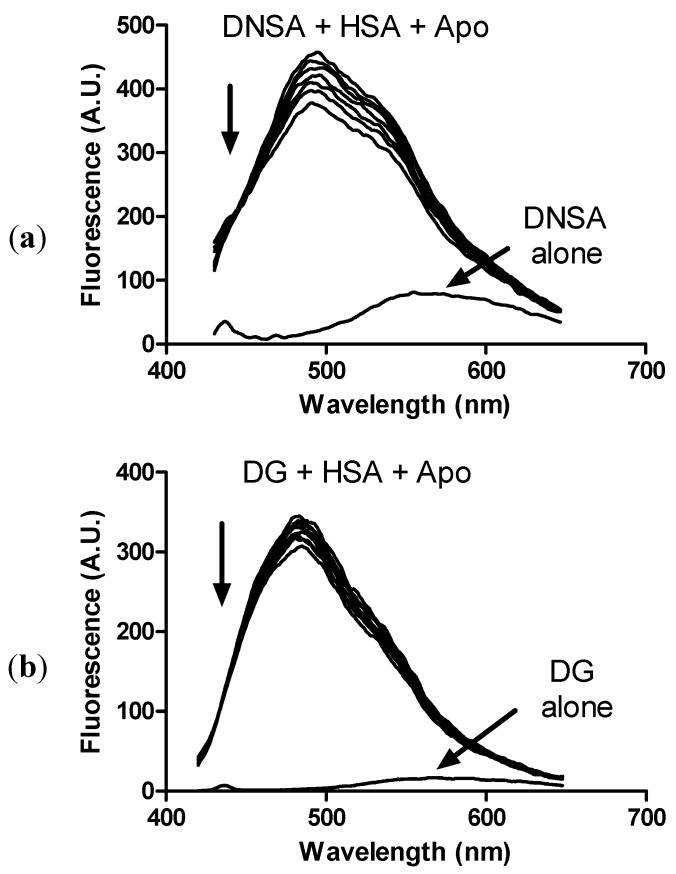
Displacement of fluorophores from HSA by apocynin. (**a**) Displacement of dansylamide (DNSA); (**b**) Displacement of dansylglycine (DG). The mixtures consisted of 10.0 μM HSA, 5.0 μM fluorophores and apocynin (0, 5, 10, 20, 30, 40 and 50 μM) in 10 mM PBS, pH 7.2 at 25 °C.

Aromatic compounds are usually bound to subdomains IIA and IIIA, site I and II, respectively, in albumin. This is the case of salicylic acid [38], which is structurally similar to apocynin. Hence, we tested the capacity of apocynin to displace fluorescent dyes which are well-characterized ligands of site I and site II, dansylamide (DNSA) and dansylglycine (DG), respectively [39]. The binding of these probes to albumin provoked a significant increase in their fluorescence quantum yield and the maximum shifted to lower wavelengths (blue shift), which was confirmed here. From the results in Figure 10, it can be observed that apocynin was able to displace both fluorescent probes, but a greater effect was observed using DNSA, an indication that apocynin has some preference for site I of albumin.

**Figure 11:**
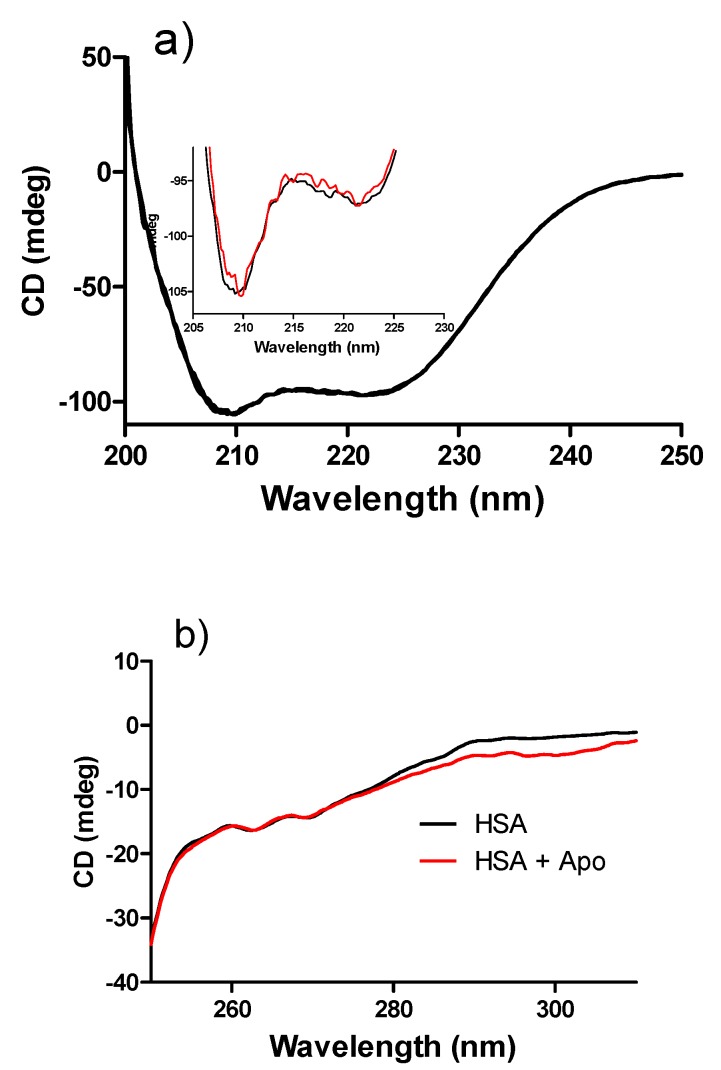
Effect of apocynin on circular dichroism spectra of HSA. (**a**) Far-UV-CD: The mixtures consisted of 1.0 μM HSA and 30 μM apocynin in 10 mM PBS, pH 7.2 at 25 °C. The inset is a zoom of graph “a”; (**b**) Near-UV-CD: The mixtures consisted of 30 μM HSA and 60 μM apocynin in 10 mM PBS, pH 7.2 at 25 °C.

In order to study the structural changes induced by the binding of apocynin to HSA, the circular dichroism spectra of the protein were measured before and after incubation with apocynin. The results depicted in Figure 11a show the typical far-UV-CD spectrum of a protein with minima at 208 and 222 nm. From these results, it can be concluded that the binding to apocynin did not alter the secondary structure of the protein. Next, the near-UV CD spectra were analyzed. The near-UV CD spectrum of albumin has two minima at 261 and 268 nm, which was also observed here. The binding to apocynin did not alter this fingerprint of the tertiary structure of HSA.

## 3. Experimental

### 3.1. Chemicals

Apocynin, protocatechuic acid, 2,2-diphenyl-1-picrylhydrazyl (DPPH), sodium nitroprusside, tung oil, Brij 35, taurine, 5,5'-tetramethylbenzidine (TMB), *N*-(1-naphthyl)ethylenediamine dihydrochloride, sulfanilic acid, horseradish peroxidase (HRP), human serum albumin free of fatty acid and globulin (HSA), 5-(dimethylamino)-1-naphthalenesulfonamide (dansylamide, DNSA), dansylglycine (DG) and 2,2'-azobis(2-amidino-propane) dihydrochloride (AAPH) were purchased from Sigma-Aldrich Chemical Co. (St. Louis, MO, USA). Hydrogen peroxide (H_2_O_2_) was prepared by diluting a 30% stock solution and calculating its concentration from its absorption at 240 nm (ε_240_ = 43.6 M^–1^ cm^–1^) [40]. Hypochlorous acid (HOCl) was prepared by diluting a concentrated commercial bleach solution and its concentration was calculated using its absorption at 292 nm (ε_292_ = 350 M^−1^ cm^−1^) [41]*.* TMB solution was prepared by dissolving 14 mM TMB and 100 μM potassium iodide in 50% dimethylformamide and 50% acetic acid (800 mM) (v/v) [41]. All reagents used for buffers and solutions were of analytical grade. HSA was dissolved in 10 mM phosphate buffer at pH 7.0 to give a 1 mM stock solution and stored at 4 °C. Accurate protein concentration was determined by measuring its absorbance at 280 nm (ε_280nm_ = 35,219 M^–1^ cm^–1^) [42] on a Perkin Elmer Lambda 35 UV−visible spectrophotometer (Shelton, CT, USA). Ultrapure Milli-Q water from Millipore (Belford, MA, USA) was used for the preparation of buffers and solutions.

### 3.2. Estimation of Logarithm of Partition Coefficient (log P)

The molecular hydrophobicity of apocynin and protocatechuic acid were calculated based on their log P values (partitioning coefficient in n-octanol/water) based on Crippen’s fragmentation method and performed using ChemDraw software (ChemDraw Ultra 7.0.1, CambridgeSoft, Cambridge, MA, USA) [25].

### 3.3. Cyclic Voltammetry Measurements

The oxidation potentials of apocynin and protocatechuic acid were obtained using an Autolab PGSTAT 30 potentiostat/galvanostat (Eco-Chemie, Utrecht, The Netherlands). A 3-electrode setup cell consisted of working electrode (glassy carbon disk electrode (GC electrode, 3 mm diameter), counter electrode (platinum plate) and the reference electrode (Ag/AgCl saturated KCl at 3 M) was used for recording the voltammetric curves at room temperature. The working electrode surface was carefully polished with 0.5 μm alumina slurries before each experiment and thoroughly rinsed with distilled water. A solution of sodium phosphate buffer 0.2 M (pH = 7) was used as a supporting electrolyte. The solutions were purged with nitrogen for 5 min before recording the voltammograms. The ethanolic solutions (5 mM) of the compounds were diluted in the electrochemical cell at final concentrations of 0.1 mM using the supporting electrolyte solution. The cyclic voltammograms were recorded at a potential scan rate of 5 mV s^−1^ [43]*.*

### 3.4. Reactivity with DPPH

The relative antiradical potency of apocynin and protocatechuic acid were compared by characterizing their capacity as reducing agents of the stable free radical DPPH. The tested compounds were incubated for 30 min with 100 μM DPPH in methyl alcohol in the dark. The scavenger activity was evaluated spectrophotometrically at 517 nm, using the absorbance of unreacted DPPH radical as a control. The scavenger activity was calculated as: [(Absorbance of control − absorbance of sample)/(absorbance of control)] × 100 [44]*.*

### 3.5. Reactivity with Peroxyl Radicals: Conjugated Autoxidizable Triene Assay

These studies were performed as previously described with minor modifications. An emulsion was prepared by mixing tung oil (2.5 mg, not stripped of tocopherols) in 10 mM phosphate buffered saline pH 7.4 (PBS, 25 mL) containing 17 μM Brij 35. The solution was vigorous vortexed to produce a homogeneous emulsion. The assays were performed as follows: the tung oil suspension (50 μL) was incubated with freshly prepared 1 mM AAPH (source of peroxyl radical) in PBS at 37 °C in the absence (control) or presence of the tested compounds in the wells of a microplate for 3 h. The final reaction volume was 200 μL. The microplate was read at 5 min intervals with shaking for 5 s before the measurements. The absorbances were measured at 273 nm using a Synergy 2 Multi-Mode microplate reader (BioTek, Winooski, VT, USA). The degradation of the triene (eleostearic acid present in the tung oil) produced an absorbance versus time curve for which the area under the curve (AUC) was calculated. The curves (AUC_sample_ − AUC_control_) against the concentration of the tested compounds were raised and their slopes used as analytical parameter for relative potency as scavenger of peroxyl radicals. Since the tested substances were also able to absorb at 273 nm, their absorbances were discounted from the total absorbances of the mixtures [26].

### 3.6. Reactivity with Nitric Oxide

These studies were performed as previously described with modifications. The reaction mixtures constituted by 10 mM sodium nitroprusside and various concentrations of the tested compounds in PBS were incubated for 120 min under a 500 W incandescent lamp. The final reaction volume was 5 mL. After incubation, an aliquot (150 μL) was transferred to a microplate and Griess reagent (100 μL, 0.05% *N*-(1-naphthyl)ethylenediamine dihydrochloride, 0.5% sulfanilic acid and 2.5% phosphoric acid) and PBS (50 μL) were added. The reaction mixtures were incubated for 10 min at 25 °C and the pink color, generated by the reaction between nitrite and the Griess reagent was measured spectrophotometrically at 548 nm. A standard curve was raised for the calculation of the produced nitrite using standards of sodium nitrite in the range (6.25–150 μM) [45].

### 3.7. Reactivity with Hydrogen Peroxide

The reactivity of apocynin and protocatechuic acid with H_2_O_2_ was monitored amperometrically with a H_2_O_2_-selective electrode coupled to a Free Radical Analyzer (TRB 4100, World Precision Instruments, Sarasota, FL, USA). The reaction mixtures were constituted of 100 nM HRP, 1 mM H_2_O_2_ and 1 mM of the tested compounds in 0.1 M phosphate buffer, pH 7.4, at 25 °C.

### 3.8. Reactivity with Hypochlorous Acid

The reaction mixtures constituted of 100 μM HOCl and various concentrations of the tested compounds in PBS pH 7.4 were incubated for 30 min at 25 °C. After that, the remaining HOCl was measured using the taurine chloramine/TMB assay as follows: an aliquot of the mixture (500 μL) was mixed with taurine (500 μL, 10 mM in PBS) and incubated for additional 5 min. Then, a portion of these mixtures (200 μL) was transferred for a microplate and TMB solution (50 μL) was added and the absorbance measured at 650 nm. The reactivity of the tested compounds was measured by correlating the consumption of HOCl with the concentration of the tested compounds.

### 3.9. Fluorescence, Quenching Studies and Apparent Binding Constant Determination

The fluorescence spectra of HSA, which were automatically corrected for emission, were obtained using a Perkin Elmer LS 55 spectrofluorimeter (Shelton, CT, USA) adjusted as follows: excitation at 295 nm and emission in the range 310–450 nm. The slit widths were 2.5 nm and 10 nm for excitation and emission, respectively. A 3-mL quartz cuvette with a 10 mm path length was used in the evaluations. The fluorescence quenching experiments were performed by the addition of varying amounts of apocynin (0–40 μM, final concentrations) to 1 μM HSA in 10 mM PBS at pH 7.4 under magnetic stirring. After addition of apocynin the mixtures were incubated for 5 min at 25 °C before measurements at 295/343 nm. The fluorescence intensities were corrected for the inner filter effect caused by attenuation of the excitation end emission signal provoked by the absorption of apocynin using the equation: *F_corr_ = F_obs_ 10^(Ab ex + Ab em)/2^*, where *F_corr_* and *F_obs_* are the fluorescence intensities corrected and observed, respectively, and *Ab_ex_* and *Ab_em_* are the absorption of the mixture at excitation (295 nm) and emission wavelength (344 nm), respectively [46]. The UV-Vis experiments were performed using a Hewlett Packard 8452 Diode Array spectrophotometer (Agilent, Santa Clara, CA, USA). The synchronous fluorescence spectra were obtained by scanning simultaneously with a fixed wavelength between the excitation and emission monochromators (Δλ) of 15 or 60 nm.

### 3.10. Displacement Studies Using Fluorescent Ligands

For displacement assays using the fluorescent ligands dansylamide (DNSA) and dansylglycine (DG), the spectrofluorimeter was adjusted as follows: excitation at 380 nm and emission in the range 420–650 nm. The slit widths were 2.5 nm for excitation and 10 nm for emission wavelengths. A 3-mL quartz cuvette with a 10 mm path length and a magnetic stirrer were used in the evaluations. It is worth of note that DNSA and DG are usually excited at 340 nm [39], but here, for avoiding the inner filter interference from apocynin, these fluorescent probes were excited at 380 nm. The experiments were performed by the addition of varying amounts of apocynin (0–50 μM, final concentrations) to a mixture of 10 μM HSA and 5 μM fluorescent ligands in 10 mM PBS at pH 7.4. After addition of apocynin, the mixtures were incubated for 5 min at 25 °C before the measurements.

### 3.11. Circular Dichroism Studies

Circular Dichroism (CD) spectra were recorded with a Jasco J-815 spectropolarimeter (Jasco, Tokyo, Japan), equipped with a thermostatically controlled cell holder at 25 °C. The spectra were accumulated in duplicate. A band width of 1 nm, response time of 1 s, scanning speed of 50 nm/min and path length quartz cells of 1 mm for far-UV-CD and 1 mm for near-UV-CD were used for the measurements. The baseline (10 mM PBS) was subtracted from all measurements. Far-UV-CD spectra were recorded at a protein concentration of 1.0 μM over the wavelength range of 200–250 nm and near-UV-CD spectra were recorded at a protein concentration of 30 μM over the wavelength range of 250–320 nm. After addition of apocynin the mixtures were incubated for 30 min at 25 °C before the measurements.

## 4. Conclusions

Currently, apocynin is the most employed inhibitor of NOX and exerts many other pharmacological effects which are not directly related to the inhibition of NOX [5,6,7,8,9,10,11,12,13,14,15,16]. As a phenolic compound, its antioxidant capacity has been suggested as a mechanism for some of its biological effects. Here, we have demonstrated that apocynin is a relatively weak free radical scavenger, hence the direct antiradical activity of apocynin cannot be responsible for the myriad pharmacological effects that have been proposed for this structurally simple, but “miraculous” molecule. On the other hand, apocynin has a high capacity as a scavenger of non-radical oxidant species as HOCl and H_2_O_2_. Hence, whether a peroxidase is expressed or added to the cells/tissues under investigation, the depletion of H_2_O_2_ by apocynin could represent an additional pathway for its biological effects. This could be particularly relevant for NOX4, since this NADPH oxidase isoform, expressed in endothelial cells, kidney, smooth muscle cells, heart and pancreas, produces mainly H_2_O_2_ instead of the superoxide anion [47]. From a pharmacokinetic point of view, apocynin binds to albumin and does not provoke alterations in the secondary and tertiary structures; hence, this could represent a pathway for its distribution in the body. However, future experiments must be done for complete characterization of its pharmacokinetic parameters like bioavailability, distribution, excretion, *etc*.
